# Supplementary material for: Novel 2D and 3D Assays to Determine the Activity of Anti-Leishmanial Drugs
Source: Microorganisms. 2020 Jun 1;8(6):831. doi: 10.3390/microorganisms8060831 (PMC7356592; doi:10.3390/microorganisms8060831)
Supplement: Supplementary file 1 [file microorganisms-08-00831-s001.pdf]

Supplementary material for "Novel 2D and 3D assays to determine the activity of anti-leishmanial drugs"

Data used to produce the values of Tables 9, 10 and 11 of the main manuscript was drawn from several different experimental data sets. All data shown in this supplementary material were derived from experiments conducted using the methods described in the manuscript's materials and methods section.

Response of four different macrophage cells – iPSCs, HBMM, MBMM and THP1 cells, infected with four different parasite strains – *L. major* JISH, *L. major* mCherry, *L. amazonensis* and *L. mexicana* to amphotericin B.

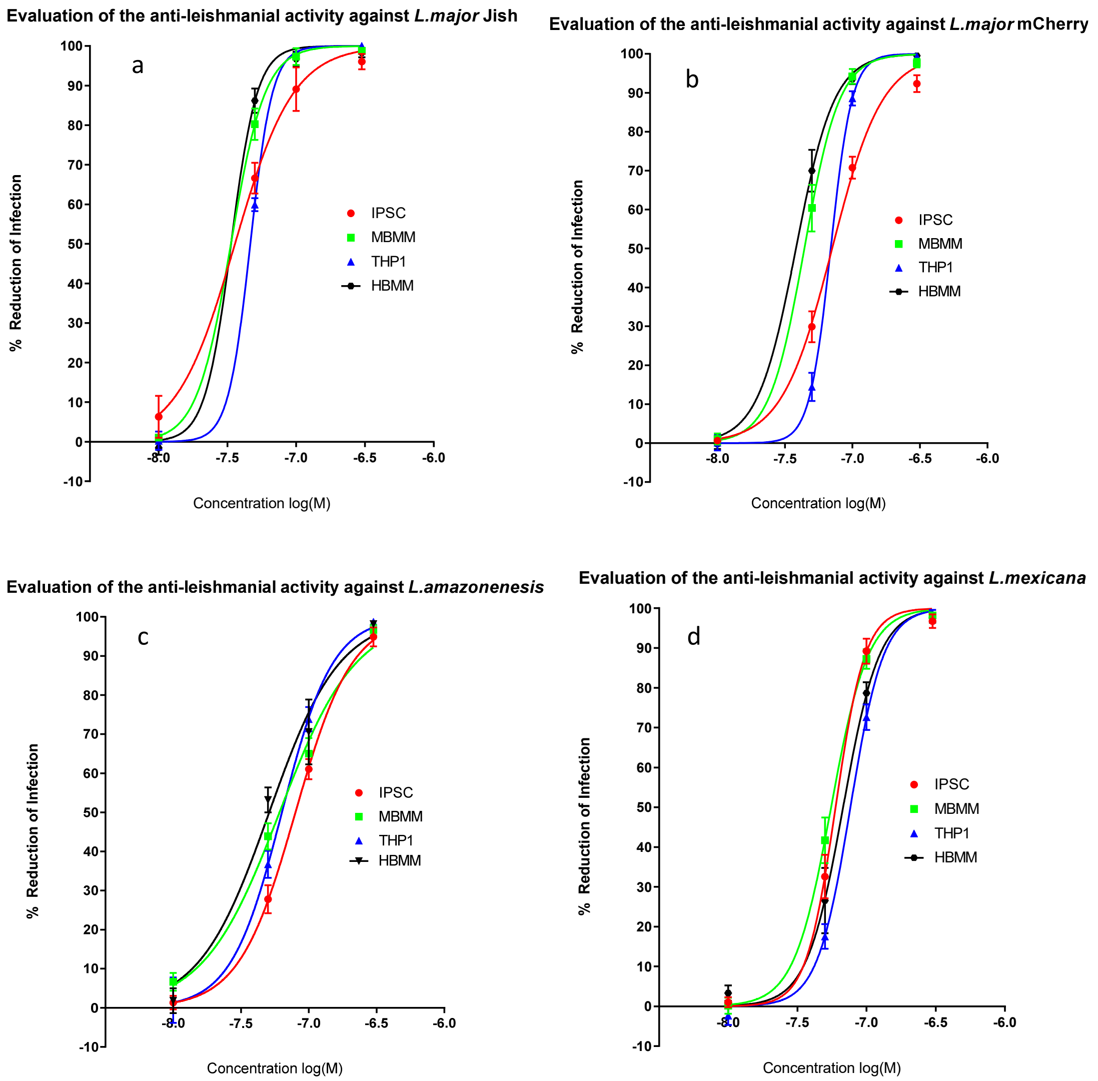

Supplementary Fig S1. Dose-response curves showing the reduction in percentage infection of four different cell types with (a) *L. major* JISH, (b) *L. major* mCherry, (c) *L. amazonensis* and (d) *L.*

mexicana produced by dosing with amphotericin b. The percentage reduction in infection calculated based on total infection seen in the untreated controls in either condition. N=9 Error bars show standard deviation.

Response of four different macrophage cells – iPSCs, HBMM, MBMM and THP1 cells, infected with four different parasite strains – *L. major* JISH, *L. major* mCherry, *L. amazonensis* and *L. mexicana* to miltefosine.

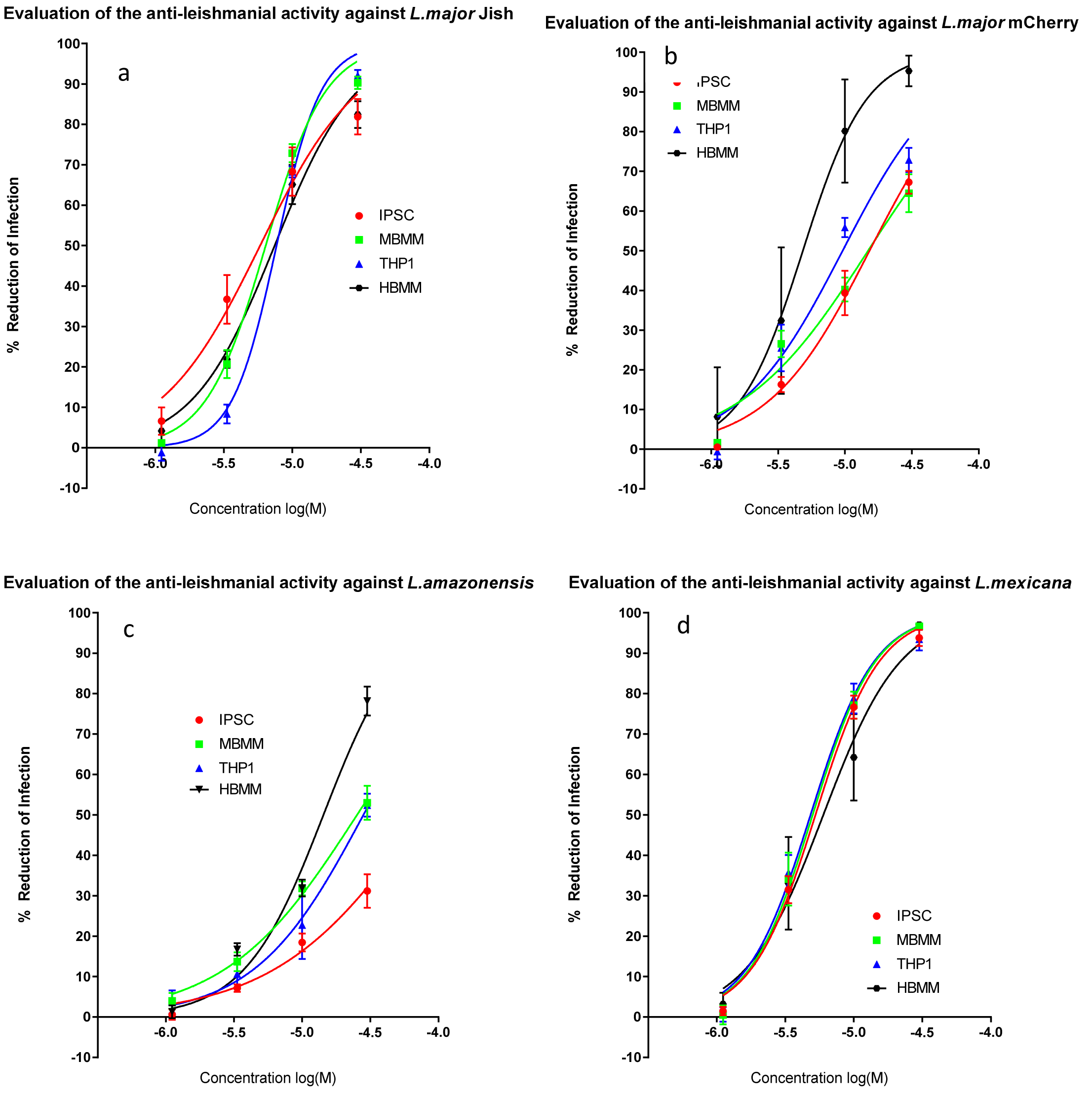

Supplementary Fig S2. Dose-response curves showing the reduction in percentage infection of four different cell types with (a) *L. major* JISH, (b) *L. major* mCherry, (c) *L. amazonensis* and (d) *L. mexicana* produced by dosing with miltefosine. The percentage reduction in infection calculated based on total infection seen in the untreated controls in either condition. N=9 Error bars show standard deviation.

Response of four different macrophage cells – iPSCs, HBMM, MBMM and THP1 cells, infected with four different parasite strains – *L. major* JISH, *L. major* mCherry, *L. amazonensis* and *L. mexicana* to sodium stibogluconate.

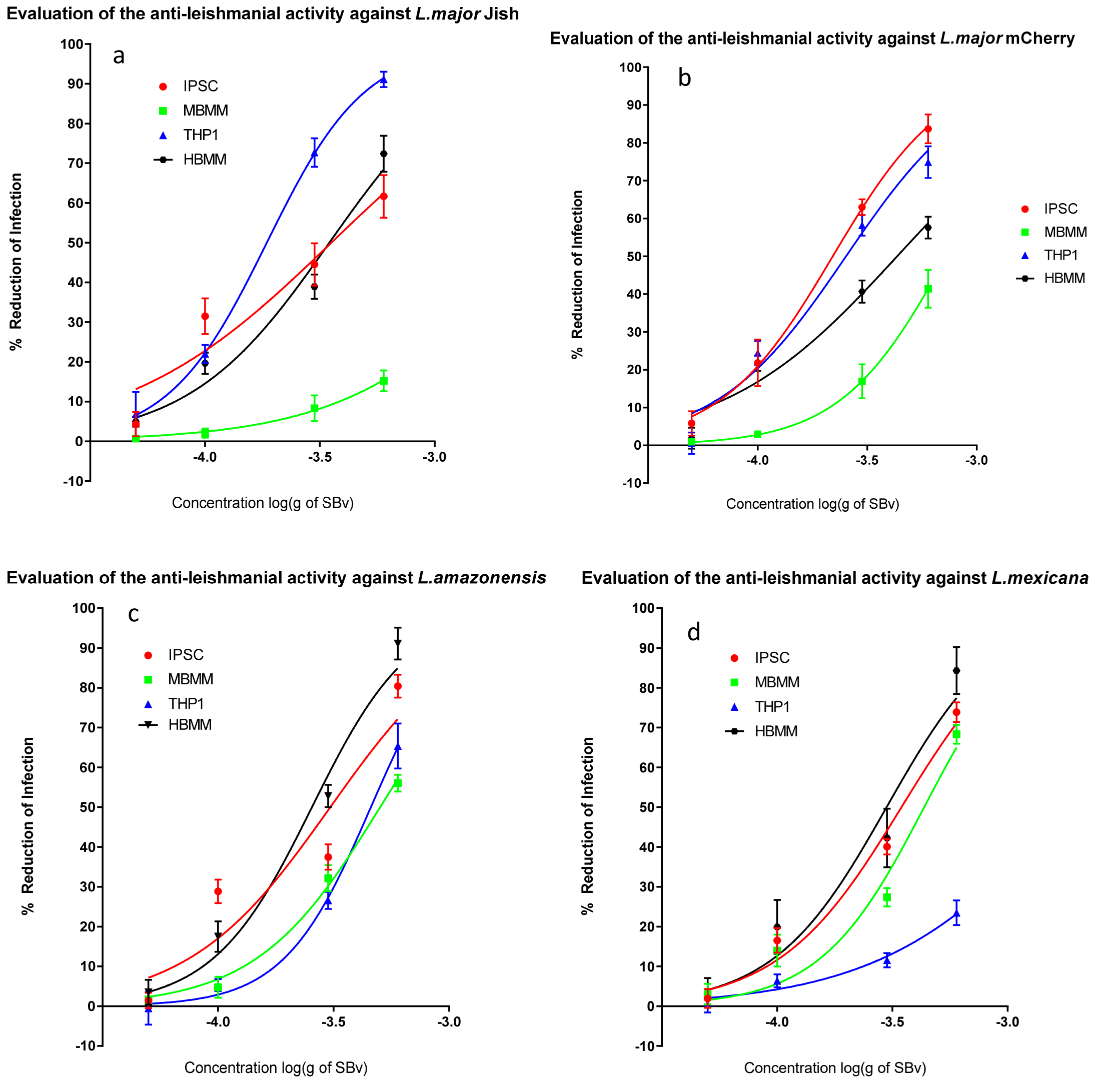

Supplementary Fig S3. Dose-response curves showing the reduction in percentage infection of four different cell types with (a) *L. major* JISH, (b) *L. major* mCherry, (c) *L. amazonensis* and (d) *L. mexicana* produced by dosing with sodium stibogluconate. The percentage reduction in infection calculated based on total infection seen in the untreated controls in either condition. N=9 Error bars show standard deviation.
